# Social learning across adolescence: A Bayesian neurocognitive perspective

**DOI:** 10.1016/j.dcn.2022.101151

**Published:** 2022-09-16

**Authors:** Lieke Hofmans, Wouter van den Bos

**Affiliations:** a Department of Developmental Psychology, University of Amsterdam, Amsterdam, the Netherlands; b Amsterdam Brain and Cognition Center, University of Amsterdam, Amsterdam, the Netherlands; c Center for Adaptive Rationality, Max Planck Institute for Human Development, Berlin, Germany

**Keywords:** Development, Adolescence, Social learning, Influence, Bayesian reinforcement learning, Uncertainty

## Abstract

Adolescence is a period of social re-orientation in which we are generally more prone to peer influence and the updating of our beliefs based on social information, also called social learning, than in any other stage of our life. However, how do we know when to use social information and whose information to use and how does this ability develop across adolescence? Here, we review the social learning literature from a behavioral, neural and computational viewpoint, focusing on the development of brain systems related to executive functioning, value-based decision-making and social cognition. We put forward a Bayesian reinforcement learning framework that incorporates social learning about value associated with particular behavior and uncertainty in our environment and experiences. We discuss how this framework can inform us about developmental changes in social learning, including how the assessment of uncertainty and the ability to adaptively discriminate between information from different social sources change across adolescence. By combining reward-based decision-making in the domains of both informational and normative influence, this framework explains both negative and positive social peer influence in adolescence.

## 1 Introduction

Adolescence is a period in which we are generally more prone to peer influence than in any other stage of our life (Nelson et al., 2005, Somerville, 2013). This increased sensitivity to peer influence is often viewed as a negative inclination: Adolescents are more likely to engage in risky behavior, such as binge drinking, smoking, or skipping school classes (Steinberg, 2007, Windle et al., 2008), especially in the company of their peers (Albert et al., 2013). However, the peer environment might also bring opportunities for the individual, as their peers can be a source of new information and guidance, such as how to adjust to a new school environment, by starting a study group, or by demonstrating kind and supportive behavior (Blakemore, 2010, Telzer et al., 2018, Duell and Steinberg, 2019, Crone and Achterberg, 2022). Copying other’s behavior or updating beliefs based on social information, also called social learning, is a hallmark of human behavior. Imitating others and learning from their rewards and punishments circumvents the need for costly and slower trial-and-error learning. To what extent we use social information depends on the situation we find ourselves in and on the characteristics of the people who are available to learn from. For example, it might be more useful to rely on other people’s behavior when you find yourself in a new situation, such as an unfamiliar country, compared to when you are at home. Social learning provides a framework that goes beyond understanding peer influence as being either good or bad for the individual, by providing deeper insights into the mechanisms of social information use.

How do we know when to use social information and whose information to use and how does this ability develop across our lifespan? Here, we review the social learning literature by focusing on the brain-behavior systems related to executive functioning, value-based decision-making and social cognition that are thought to underly social learning. Where needed we will turn to the adult literature to inform our predictions, but we place a special emphasis on adolescence. Adolescence is a period of strong social reorientation, during which peers become more important (Nelson et al., 2005), and brain areas involved in social learning, both structurally and functionally, develop markedly during this period (Blakemore, 2008, Blakemore and Mills, 2014, Mills et al., 2014, Kilford et al., 2016, Andrews et al., 2021). Apart from a behavioral and neural viewpoint, we will also review the literature from a computational viewpoint and will introduce a Bayesian reinforcement learning framework to make testable predictions about developmental changes in social learning. Compared with verbal models, computational models have the advantage of quantifying detailed predictions about behavior and brain activity and providing access to the latent cognitive processes that compose observed behavior. This level of specificity allows for rigorous testing and falsification, thereby being a promising approach to improve inferences about developmental trajectories (Forstmann et al., 2011, Cheong et al., 2017, van den Bos et al., 2018).

## 2 Computational framework

### 2.1 Reinforcement learning

Here we will sketch out the contours of a computational framework for understanding (developmental changes in) social learning in adolescence. Although an exhaustive formalization of such a model is beyond the scope of the current review, we provide a formalization of the basic mechanisms that can be adapted and applied to different social tasks and contexts (e.g. risky and prosocial behavior). For examples of more detailed descriptions of formal social learning models, we refer the reader to (Ciranka and van den Bos, 2019, FeldmanHall and Nassar, 2021).

**Fig. 1:**
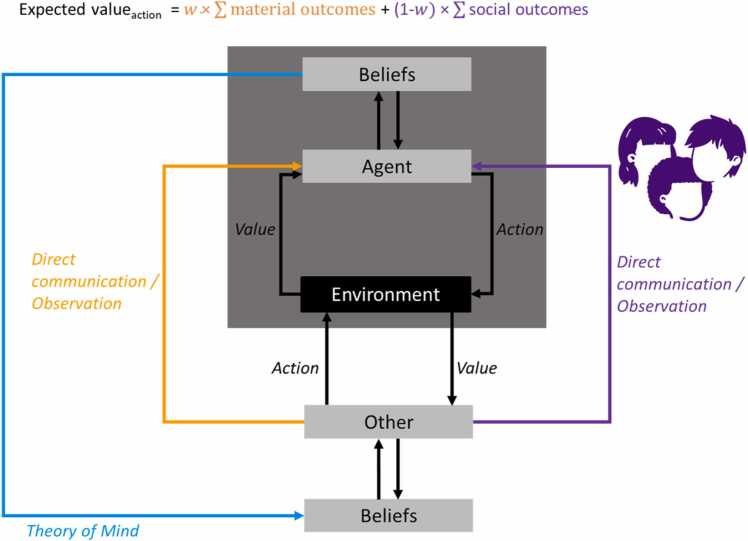
Social learning model. An agent learns through non-social informational reinforcement learning (dark grey box), in which they choose the action that is believed to render the highest expected value in terms of material reward and punishment such as food, energy, money or pain. This action results in outcomes that can be used to update beliefs about the value function (Eq. 1). Social learning involves learning about actions and outcomes by direct communication or observation of others. On the one hand, social learning involves informational reinforcement learning (orange) analogous to non-social learning. On the other hand, social learning involves normative reinforcement learning (purple) to pursue social rewards such as a sense of belonging, status or friendship, or to avoid punishment such as social exclusion. Both expected material and social outcomes are updated in a Bayesian fashion and combined into an overall expected value function. The weight an agent assigns to informational versus social outcomes (w) depends on the individual, the context and the task. For example, social outcomes might only be relevant in the presence of a particular social group. Theory of Mind (blue) is when we infer someone else’s goals and beliefs using the set of actions they performed and the outcomes they received in the past, to ultimately decide to what extent we want to use the other’s social information to update our own beliefs.

To formulate a computational account of social learning, we will start from the viewpoint of a standard reinforcement learning (RL) framework (Fig. 1, dark grey box), in which an agent learns the value of specific actions by using reward prediction errors (Schultz et al., 1997, Sutton and Barto, 1998). A prediction error is the difference between what an agent expects after a certain action in a certain situation and what they ultimately observe, such as whether or not you are invited to a party after you befriended the host, or whether or not your soup turns out alright after following certain instructions. More precisely, a specific action will be reinforced when an agent receives a higher net reward than previously expected, and will be discouraged when a lower than expected net reward is received. This can be formalized according to a Rescorla-Wagner rule (Rescorla and Wagner, 1972):

(1) $${\hat{Q}}_{t+1}={\hat{Q}}_{t}+\alpha \times (Q_{t}-{\hat{Q}}_{t})$$

where the expected value of an action $\hat{Q}\;$ at timepoint t is updated to accommodate the difference between the actual value at timepoint t ($Q_{t}$) and the expected value (i.e. the prediction error $\left(Q_{t}-{\hat{Q}}_{t}\right)$), to arrive at a newly computed expected value at the next timepoint t + 1. The rate at which the expected value of an action gets updated in response to the newly observed value depends on the learning rate α, which is higher if the agent puts more emphasis on the new observation relative to previous observations.

The actual value, and by extension the expected value, is the sum of all received benefits minus all costs in response to performing an action. It is therefore essential to regard multiple sources of benefits and costs associated with one action. Benefits can include material rewards, such as receiving a salary in return for work, self-fulfillment or a feeling of happiness after successfully completing a difficult puzzle, new information, but also social rewards such as social acceptance after adhering to group norms. Costs can be material such as financial costs to for example get your car repaired after foolish behavior on the road, physical discomfort after a car collision, or they can be of a social nature, such as social exclusion after spreading gossip about a friend or not adhering to group norms.

We define these action-value pairs loosely as a set of beliefs, or a model that can be used to select the action with the highest expected value. Of note, our model is agnostic as to whether the updating of these beliefs is a process of which the agent is aware.

### 2.2 Bayesian reinforcement learning

**Fig. 2:**
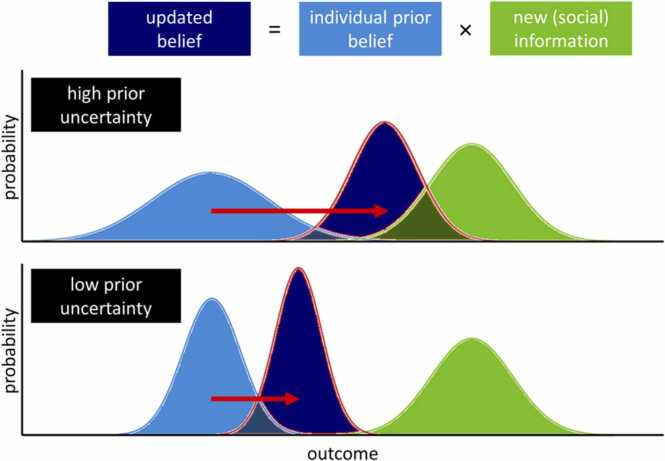
Bayesian updating under uncertainty. An agent combines their prior belief or information (light blue) with new information (green) to arrive at an updated belief of the expected value or outcome (dark blue). Higher uncertainty is represented as wider distributions. In the case of high prior uncertainty (top panel), the prior information is assigned a relatively low weight, resulting in a stronger impact of the new information (red arrow). Under low prior uncertainty (bottom panel), the prior information is assigned a relatively high weight, resulting in a less pronounced impact of the same new information.

Estimating all expected benefits and costs involves various forms of uncertainty (Bach and Dolan, 2012, Yon and Frith, 2021). First, because the range of sources of all benefits and costs is so extensive, not all of them can be considered or foreseen. Second, the outcomes of our actions are stochastic because we hardly ever find ourselves in exactly the same situation, making it hard to predict the consequences of our behavior. Classic Rescorla-Wagner types of RL models are not able to incorporate uncertainty. In contrast, Bayesian learning models capture uncertainty by computing a probability distribution over outcome beliefs (Beck et al., 2008, Gershman, 2015, Gershman and Uchida, 2019). This means that the expected value of a certain action is not a point estimate, as it is for classic RL, but rather a probability distribution over a range of outcomes. The shape of this probability distribution depends on our prior experience and is updated, by Bayes rule, when new information is coming in. Importantly, the effect of new information on changes in beliefs is dependent on the strength of the prior beliefs: When there is a very weak prior, in other words someone is very uncertain about the state of the world, new information will have more impact compared to someone who is very certain and receives the same information (Fig. 2). In the case of preparing a soup, you might not be completely sure about the chance that your soup comes out right. Therefore, you might be inclined to incorporate someone else’s advice on how to improve your soup. In contrast, a professional cook with years of experience in preparing this soup will trust their own skills and will be less keen to follow the advice. Thus, in the case of a soup and with every potential action, you need to combine prior knowledge and new incoming information to arrive at a probability distribution of how well you think an action will turn out and this probability distribution is continuously updated with every new piece of incoming information. Decreases in how adolescents experience uncertainty will be especially important in understanding developmental differences in social learning, since social information use is likely to be lower when uncertainty decreases, as we will point out in Section 3.

### 2.3 Social reinforcement learning

Before discussing social learning or the use of information from social sources, we need to acknowledge the differences between informational and normative influence (see also Deutsch and Gerard, 1955). Informational social influence involves the updating of factual beliefs based on information from others, including the formation or updating of decisions derived from those beliefs (Deutsch and Gerard, 1955, Cialdini and Goldstein, 2004). For example, when deciding on whether to wear an extra jacket today to protect you from the cold, one might base their decision on what they see other people wearing outside or based on the advice from their mother. Normative social influence, on the other hand, is motivated by anticipated social reward and punishment, such as social inclusion in or exclusion from a group (Deutsch and Gerard, 1955, Cialdini and Goldstein, 2004). While normative might refer to typical as opposed to abnormal or clinical in some contexts, we here refer to behavior that is common for or approved of by others. Normative influence or learning about normative behavior in the current context involves how others’ behavior leads us to conform in order to acquire social benefits or avoid social costs. For example, while aggressive behavior might be normative within a group of young delinquents and displaying this behavior might lead to social, or normative, gain within that group, it is certainly not adaptive or typically displayed by youths in general. Social influence thus involves the updating of beliefs that pertain to the way people think they ought to behave, or the extent to which they adhere to social expectations due to perceived social pressure stemming from either a group or a single other person, and is therefore heavily dependent on the specific social environment (Hogg and Reid, 2006). In many decisions, both informational and normative influence play a role and both material outcomes stemming from informational influence and social outcomes stemming from normative influence are incorporated into the value function in a Bayesian fashion (Fig. 1).

#### 2.3.1 Social reinforcement learning: informational influence

Using social information can reduce uncertainty and speed up the learning process by incorporating the observed material benefits and costs that were experienced by others, rather than yourself, into your expected value function (Fig. 1, in orange). For example, if you see you sister adding one pinch of salt to her soup, tasting it, grimacing, and adding another pinch of salt, you might infer that only one pinch of salt was too little. Besides observing others’ actions and associated outcomes, someone else might also simply tell or advise you what to do (e.g. the recipe may tell you to add two pinches of salt). This informational influence of others can be incorporated into the computational model by a simple initial updating step. Imagine the following scenario: You are going to a new soup restaurant that specializes in novel soups. On the menu you find all kinds of soups that you have never tried before, and they all look equally appealing, so their priors are the same. Because you do not know what to choose you ask your friend, who is a soup connoisseur and frequently visits this restaurant, for advice. What happens if she recommends the bisque is that now your expectation for that soup increases, but that for the others stays the same as before. Next, you might indeed order the truffle soup and taste it yourself. You may for instance like the soup, thereby strengthening its value. Alternatively, you might not be very fond of it, leading to a large negative prediction error that updates your expected value of the soup such that now it falls below the expected value for the others soups and making it far less likely that your will order it again. Thus, informational social information enters the expected value function in the same way as any self-experienced piece of information (although the strength of the social information may depend on the expertise of the person giving the recommendation, as discussed in Section 5). However, it is also conceivable that you do believe your friend knows very little of soups but that you just follow her advice to be nice and not violate social customs, which brings us to normative influence.

#### 2.3.2 Social reinforcement learning: normative influence

Normative social influence depends on the behavioral expectations held by another person or a particular social group. One can learn about these norms or expectations by the observation of others or by direct communication (Fig. 1, in purple). For example, you and your new group of friends visit a soup restaurant. You notice that everyone orders a vegetarian soup, so you infer that the group norm is to eat vegetarian. In contrast, someone might also simply tell you that most group members eat vegetarian. This normative information results in a higher expected value for a vegetarian soup and lower values for meat- or fish-based soups, because you want to fit into the group and you don’t want your soup preferences to be frowned upon by your new friends. Importantly, this group norm is only relevant in the presence of that specific group. For example, even though the majority of the group eats vegetarian, one of the group members might really like the bisque. The next time you visit the restaurant with that person, you might still order the bisque to maximize social value, which would also be an instance of normative influence. Expected value related to social benefits and costs can be updated in a Bayesian manner, analogous to expected material value, to account for the uncertainty that stems from hidden preferences and norms that are needed to be inferred from the behavior shown by group members. Moreover, this updating can take the form of an instantaneous increase or decrease of the expected value of an action, such as when the social norm or someone else’s preference is clearly communicated, but the updating can also be repetitive, such as when you need to learn about someone’s soup preference by trial and error.

Note that informational and normative social influence are not mutually exclusive, as one might initially display behavior merely to conform to the group norm and to avoid social punishment, but later actually incorporate this behavior into their own norms. The key difference, however, between informational and normative social influence is that normative influence is driven by the desire to be accepted by a particular social group (Cialdini and Goldstein, 2004), whereas informational influence is motivated by the formation of accurate representations of reality. Normative and informational influence can therefore also work in opposite directions. Take the example of social risk: Even though you have learned that the bisque is your favorite soup on the menu, you might still opt for a vegetarian soup when you are with your vegetarian friends, because you do not want to risk social rejection. Or, while you have observed that driving under the influence of alcohol is dangerous, the group norm might be to behave risky, which nevertheless prompts you to also behave in a risky way and drive under influence to impress others (see also (Vives et al., 2022)). Thus, normative influence is particularly pertinent in situations where behavior is observed by peers (Deutsch and Gerard, 1955) and social reward or punishment are more imminent (Liu et al., 2017). We note that in this review, we focus on how adhering to group norms or pleasing others is a means to maximize subsequent social reward for the self, rather than on how maximizing benefits for others such as in prosocial learning (Lockwood et al., 2016, Westhoff et al., 2021) can be a goal in and of itself, which is outside the scope of the current review (but see Theriault et al., 2021).

Even in the face of conflicting new informational evidence, normative influence can retain its impact, which is intuitive when we think about how norms might bias our decisions. Changes in how adolescents weigh informational versus normative influence (Section 4) and how they weigh different social sources (Section 5) are important determinants of development of social learning.

The framework we sketched above can give us more insight into the developmental mechanisms of peer influence. We aim to disentangle the different elements that may play a role in the sensitivity to peers as is often observed, which can result from adolescents experiencing more uncertainty, adolescents being more motivated to conform to the group, or adolescent changes in their ability to differentiate between the usefulness of different social sources. These elements are all subject to change over the course of adolescence, when brain areas and social structures in the environment strongly develop.

## 3 Informational peer influence in adolescence: the role of uncertainty

The way in which we assess uncertainty and how we deal with uncertainty is important for our subsequent decisions and how we incorporate social information. Here, we will lay out how adolescents’ appraisal of uncertainty differs from that of other age groups, how this depends on the development of different brain areas, and how this might lead to differences in social information use. In doing so, we will focus on the informational value of others’ behavior or advice in order to resolve uncertainty and inform our expectations.

### 3.1 Adolescents show reduced ambiguity aversion

Firstly, we note that when talking about risk in this context, we mean informational risk, rather than for example the social risk of being judged by your peers. Several studies have investigated how the appraisal of risk and uncertainty in adolescence differs from that in other age groups. It has been shown that the ability to judge probabilities when they are explicitly known are matured by mid-adolescence. In a gambling task, children (5–8 year old) underweight the probability of low-probability events and overweight the probability of high-probability events, a tendency that decreases with age, with adolescents of 14–20 year old being almost at par with adults (21 + year old) (Harbaugh et al., 2002). Another study in participants between 8 and 30 year old revealed that the ability to estimate probabilities during a gambling task did not differ between children, adolescents and adults (van Leijenhorst et al., 2008). Thus, the ability to judge known probabilities cannot explain adolescent increases in for example real-life risk behavior or the copying of peer-behavior. In this regard, it is important to note the difference in uncertainty between risk and ambiguity. Whereas risk requires a consideration between options with known probabilities but with a chance-based outcome, ambiguity requires a consideration between options with unknown probabilities. It might well be that ambiguity, but not risk judgments differ between adolescents and other age groups. A study that compared ambiguity aversion between children (8–9 year old) and adults (19–27 year old) in which participants played a gambling task found significant ambiguity aversion in adults, but not in children, such that adults but not children preferred a risky gamble with known probabilities over an ambiguous gamble with unknown probabilities (Li et al., 2015). Another study (Tymula et al., 2012), which was replicated by (Blankenstein et al., 2016), revealed an increased willingness to choose an ambiguous lottery over a safe bet for adolescents compared with adults, an effect that was stronger for higher ambiguity levels, with higher levels of ambiguity aversion additionally correlating negatively with real-life risk-taking (Blankenstein et al., 2016). Interestingly enough, in both studies adolescents’ risk aversion did not differ from that of adults. In line with these results, a later study found that adolescents are more accepting of uncertainty and ambiguity than children and adults, which was again related to self-reported real-life risk taking, whereas risk aversion declined monotonically over age and was not related to real-life risk taking (van den Bos and Hertwig, 2017). These results suggest that the often observed increase in risk engagement in adolescents is due to an increased willingness to gamble when they lack complete knowledge, rather than increased risk-taking per se (but see Niebaum et al., 2022). This reduced ambiguity aversion might well be adaptive during adolescence, because adolescents relatively often find themselves in new, uncertain environments, in which behavior that promotes exploration can boost learning (Ciranka and van den Bos, 2021a).

### 3.2 Adolescents experience higher uncertainty

Apart from adolescents often finding themselves in new and therefore relatively uncertain situations, it is an interesting but as of yet open question whether adolescents also perceive uncertainty differently from other age groups. Indeed, a higher perceived uncertainty in adolescents compared with adults is supported by studies using reinforcement learning paradigms. For example, using a probabilistic reversal learning task in which participants needed to adapt to changes in reward contingencies, adolescents showed less certainty in their decision-making than adults (Javadi et al., 2014). The authors speculate that this relates to a less accurate model of the current state or environment, as a result of a less optimal updating process. This hypothesis is substantiated by a study demonstrating that in a noisy but stable environment adolescents show a smaller decrease in learning rate and exploration over time and have lower choice accuracy than adults, implying that they might overestimate the volatility of the environment, corresponding to weaker priors and a less precise estimate of the current state (Jepma et al., 2020).

### 3.3 Higher uncertainty leads to more social information use

**Fig. 3:**
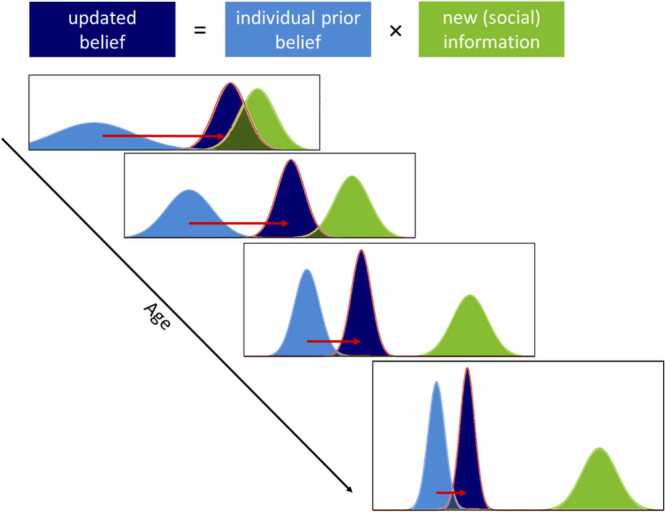
Experienced uncertainty as a function of adolescent age. In general, experienced uncertainty about prior beliefs tends to decrease over the course of adolescence (narrower curves in light blue), resulting in older adolescents being influenced by others to a lesser extent than younger adolescents (red arrows).

These weaker priors or more uncertain expectations in adolescents compared with adults might lead to further information seeking to resolve this uncertainty. A recent study found that, using an effort-based information sampling task, adolescents sampled more information than young adults before reaching a decision (Niebaum et al., 2022). Taking this to the social domain by using a risky decision paradigm in which uncertainty and the presence of social information were manipulated, Ciranka and van den Bos found that young adults used the choices of others to a larger extent when they were more uncertain (Ciranka and van den Bos, 2020). Participants had to choose between a higher but potential reward (risky option) or a lower but fixed reward (safe option), either with or without viewing the option chosen by another player. In the certain condition, participants were explicitly told the probability to win the potential reward, whereas in the uncertain condition, participants had to learn about the probability by being provided with sequential information about the probability. Using a Bayesian updating model, the authors showed that participants changed their choice preference more often in the direction of the choice made by the other player in the uncertain versus the certain condition and when they were subjectively more uncertain within conditions. Moreover, a recent study in which adolescents had to decide whom to trust revealed an adolescence-emerging increase in uncertainty of prior beliefs, which accounted for differences in information sampling about others' trustworthiness (Ma et al., 2022). In another study by Moutoussis and colleagues, a group of 14–24 year olds played a temporal discounting task in which they could choose between a smaller but immediate reward or a larger but delayed reward, and they indicated their preferences both before and after viewing the preferences of others (Moutoussis et al., 2016). Changes in preferences from before to after viewing the social information correlated with participant’s choice variability at the first time point, such that the higher the variability, or the less robust their initial preferences, the more they were influenced by others. In addition, younger participants were less certain about their own initial preferences and were also influenced more by others than older participants. Although normative influences cannot be excluded, these findings are in accordance with a Bayesian updating model in which the integration of new social information leads to substantial higher certainty, or a narrower curve, of the posterior than the prior. This corresponds to an added value in our computational framework, where the prior value for peer-promoted decisions, but not other decisions, receives a higher value, making that decision more valuable compared to other possible decisions (and vice versa for decisions that were discouraged by peers), thereby reducing overall uncertainty about which decision to take. Thus, adolescents seem to experience higher levels of uncertainty, driving them to use more social information (Fig. 3). Which brain areas are involved in these processes?

### 3.4 Neural mechanism of uncertainty

#### 3.4.1 Uncertainty estimates in the brain

fMRI studies using visual estimation tasks have established that sensory uncertainty or Bayesian probability distributions can already be decoded from the sensory cortex and that this decoded uncertainty correlates with behavioral choice variability (van Bergen et al., 2015, van Bergen and Jehee, 2019) and subjective confidence ratings (Geurts et al., 2022). Multiple areas have been demonstrated to play a role in different aspects of decision confidence. These include the posterior parietal cortex, tracking sensory reliability or the inverse of sensory noise (Bang and Fleming, 2018), the ventromedial prefrontal cortex and the perigenual anterior cingulate cortex, tracking subjective confidence (De Martino et al., 2013, Lebreton et al., 2015, Bang and Fleming, 2018, Gherman and Philiastides, 2018), and the ventral striatum, tracking sensory reliability (Diederen et al., 2016, Bang and Fleming, 2018) and confidence ratings (Hebart et al., 2016). Moreover, connectivity between the rostrolateral and ventrolateral PFC with the ventral striatum have been suggested to transform representations of decisions into representation of confidence in that decision (De Martino et al., 2013, Hebart et al., 2016), thereby being important for a Bayesian style of decision making.

#### 3.4.2 Neural correlates of information seeking

Extending these findings to behavioral correlates, Kaanders and colleagues investigated the neural mechanisms of how much information adult participants sampled before committing to a decision between two sets of stimuli (Kaanders et al., 2021). They found that the medial frontal cortex encoded choice difficulty, but that this effect disappeared when including whether or not participants would sample more information before making a choice, suggesting that choice difficulty is an important factor in determining whether or not to search for more information. This corroborates earlier findings where activation in both the insula and medial frontal cortex was higher for instances in which participants chose to observe a gamble versus instances in which they chose to bet themselves (Blanchard and Gershman, 2018), indicating that these regions play a critical role in the exploration of additional information. Conversely, activation in the ventromedial PFC correlated negatively with updating of predictions, implying that this area encodes to what extent there is a match between predictions and outcomes, which might in turn signal to stop exploration. It is thus crucial to distinguish between different functions for different subparts of the medial frontal cortex.

#### 3.4.3 Maturation of the executive network and uncertainty during adolescence

Thus, sensory uncertainty is already encoded in early sensory areas and in higher cognitive areas such as the posterior parietal cortex and medial (pre)frontal cortex, with activation in these areas correlating with confidence ratings and information seeking. Connections between more anterior lateral areas of the PFC and striatal areas seem to be important for awareness of uncertainty. The question arises how the relatively late development of these more cortical areas (Gogtay et al., 2004, Casey et al., 2008, Casey, 2015) impacts uncertainty, confidence ratings and information seeking during adolescence.

As discussed above, adolescents seem more uncertain in their learning and decision making and tend to seek out additional information to inform their decisions. Many open questions remain. Might it be the case that, while sensory precision can already be decoded in sensory brain areas, this is not translated to the more parietal and prefrontal cortical areas because they are still in development (Gogtay et al., 2004, Casey et al., 2008, Casey, 2015), rendering adolescents less adept at using precision estimates and therefore more uncertain in their learning and decision making? Does the degree of maturation of the higher cognitive areas at the individual level correspond to lower uncertainty in adolescents (De Neys et al., 2011; Reiter et al., 2021)? Is the combination of higher uncertainty and tolerance for ambiguity related to less reliable confidence estimates in adolescence? Perhaps most important in the context of the current review: How does uncertainty of the environment affect adolescents’ social information use and how does this compare to the effect in adults, given adolescents’ lower seemingly lower sensitivity to different degrees of uncertainty but their overall higher experienced uncertainty? We could for example hypothesize that adolescents use more social information than adults on average, but show smaller differences as a function of how uncertain the environment is. How does neural activation in response to uncertainty change over the course of adolescence and do these neural patterns correlate with the extent of social information use, such that sharper neural distinctions between low and high uncertainty lead to clearer differences in social information use?

It is thus not yet clear how developmental changes in how uncertainty is assessed at the neural level lead to differences in social information use, although it has been established that higher uncertainty leads to more social information use. In the next section, we will shift focus to a more normative rather than informational role of social cues. We will then discuss how adolescents differentiate between social information and how this alters the extent of social information use.

## 4 Normative peer influence in adolescence

### 4.1 Increased normative influence in adolescence

Adolescence is a period of strong changes in socially motivated behavior. It has often been demonstrated that compared with other age groups, adolescents experience stronger peer influence (Albert et al., 2013, Telzer et al., 2018). Whether this increased peer influence is negative or positive appears to be context-dependent and modulated by the norm within the peer group. (Choukas-Bradley et al., 2015, van Hoorn et al., 2016, Chierchia et al., 2020, da Silva Pinho et al., 2021, Molleman et al., 2021). For instance, adolescents show increased risk-taking when they are being observed by peers versus when they played the game alone while playing a simulated driving task (Gardner and Steinberg, 2005, Chein et al., 2011) or a gambling task (AR Smith et al., 2014), but also increased cognitive control when playing a Go/No Go task on behalf of a high compared with a low status school mate (Sharp et al., 2022) and increased prosocial behavior after having received prosocial feedback from a peer (van Hoorn et al., 2016). Crucially, a study using a simulated driving task in which the peer norm was manipulated such that peer passengers were either risk-accepting or risk-averse showed that adolescents exhibited a riskier driving style when in the presence of a risk-accepting passenger, but took fewer risks when in the presence of a risk-averse passenger, compared with driving alone (Bingham et al., 2016).

These peer effects presumably stem from anticipated social rewards or avoidance of social punishments. This susceptibility to social acceptance and rejection is particularly strong during adolescence (Somerville, 2013, Foulkes and Blakemore, 2016, Blakemore, 2018, Andrews et al., 2021), possibly because of the social reorientation during this period and because they have to establish a status within a new social group (Crone and Dahl, 2012, Blakemore and Mills, 2014). Evidence for such a cognitive hypersensitivity to valenced social stimuli during adolescence comes from studies investigating the processing of socio-emotional stimuli during a cognitive control task. Children, adolescents and adults played a task in which they either had to respond to a target (Go) or inhibit their response to a non-target (No-Go) with both appetitive cues (happy faces) and neutral cues (calm faces) (Somerville et al., 2011). Impulse control to neutral cues showed improvement over age, but failure to inhibit responses on appetitive No-Go trials was increased in adolescents but not in children and adults. In a follow-up study, the authors used the same paradigm but now analyzed failures of impulse control for fearful faces (Dreyfuss et al., 2014). They found that compared with children and adults, adolescents made more errors on fearful versus calm No-Go trials.

This heightened susceptibility to peer norms fits into the social RL framework as a stronger impact or a narrower curve of normative peer information for adolescents compared with other age groups, shifting the balance away from your individual prior or from, perhaps conflicting, informational influence. The mean of this normative information can either be more negative than your initial personal expected value of a particular action, steering you away from that action to avoid social punishment, or it can be more positive, encouraging to perform that action to pursue social acceptance. It is of note that the influence of encouraging versus discouraging peer influence might also show different developmental trajectories, as learning from positive versus negative feedback has been shown to be increased in adolescents compared with children and adults leading to enhanced approach behavior (Jones et al., 2014, Silva et al., 2016, Somerville et al., 2010, van den Bos et al., 2012), raising the hypothesis that the effect of norms that promote particular behavior is greater than that of norms that discourage behavior. Testing the hypothesis that adolescents indeed experience a higher sensitivity to normative information would require formulating a Bayesian model of social influence to test how and to what extent adolescents, compared with other age groups, integrate the new social information with their own prior information. In addition to behavioral experiments, neuroimaging studies could inform us about the neurocognitive mechanisms that lay at the base of these computational mechanisms of the integration of normative information, as we will discuss next.

### 4.2 Neural mechanisms of increased normative influence

Studies using fMRI have established that neural activity in reward-related areas, including the ventral striatum and the orbitofrontal cortex, shows a greater increase in adolescents than children and adults while playing a game in the presence of peers compared when playing the game alone (Chein et al., 2011, Smith et al., 2015) or when expecting positive social reinforcement (Jones et al., 2014). Another study investigating the effect of both positive and negative social incentives on reaction times (social incentive delay task) demonstrated that the anticipation of social reward and punishment both led to stronger ventral striatal activity, which was accompanied by faster reaction times (Kohls et al., 2013). Moreover, an adolescent-specific failure to inhibit responses to happy and fearful versus calm faces was accompanied by increased neural activity in the ventral striatum (Somerville et al., 2011) combined with the orbitofrontal and medial prefrontal cortex (Dreyfuss et al., 2014), respectively. It has been suggested that this increased responsiveness results from the neural maturation of these brain areas (Somerville et al., 2010, Blakemore and Robbins, 2012, Crone and Dahl, 2012, Albert et al., 2013) and changing neurochemical transmission including elevated release of striatal dopamine in response to reward (Galvan, 2010, Luciana et al., 2012). Importantly, studies that index pubertal status by measuring hormone levels have found that gonadal hormone levels correlate with reward-related activity in the ventral striatum (Op De Macks et al., 2011, Braams et al., 2015), connectivity within the social brain network including the temporoparietal junction and the dorsomedial PFC (Klapwijk et al., 2013), and willingness to sacrifice money for social status (Cardoos et al., 2017), suggesting more directly that the adolescent-specific increase in social responsiveness is related to developmental status (Nelson et al., 2005, Blakemore, 2008, Crone and Dahl, 2012). In terms of the Bayesian framework, this would predict that parameters that govern the relative weight of normative influence would play an outsized role in adolescence. Future studies that include hormonal measures could also further explore which model parameters correlate with hormones to get a better handle at their impact on changes in adolescent behaviors (Laube et al., 2017).

In addition to a mere increase in activation of reward-related areas, it has been argued that especially stronger neural connections between reward-related areas and decision and control areas might lead to stronger behavioral effects of social stimuli. For example, it has been found that over the course of adolescence, there is a strengthened connectivity between the reward-related ventral striatum and the medial prefrontal cortex, which is implicated in social cognition (Amodio and Frith, 2006) and has strong connections with the parieto-frontal control network (van den Bos et al., 2012). Similar results were found in a recent study in which adolescents performed a risky driving task while completing an fMRI scan, both before and after they observed an older sibling complete the task (Rogers et al., 2022). The authors found that adolescents who were more likely to be influenced by their older sibling showed more directional connectivity from the ventral striatum to the medial prefrontal cortex, possibly guiding learning that is more strongly motivated by the rewarding nature of the social cue. These studies suggests that ventral striatal reward signals might be more readily translated to value-based decision making and social learning behavior due to enhanced functional connectivity in adolescence.

In sum, normative social stimuli seem to be more readily processed during adolescence compared with childhood or adulthood due to stronger connectivity of the reward system with social and executive brain areas. This might change the total reward value of specific peer-promoted (or peer-discouraged) actions by adding (or subtracting) a normative component to the total reward value of an action, for example by anticipated enhanced status or social exclusion. However, in the context of normative social information as well as in the context of uncertainty and the search for informational social information, it is important to not blindly follow everyone but to differentiate between different sources of social information. In the next section, we will discuss how peer characteristics might differentially modulate social information use in order to resolve uncertainty or promote social reward.

## 5 From whom to use social information: social cognition in adolescence

### 5.1 Peer characteristics important for social information use

In order to optimize the way in which we use social information, we should not merely use everyone as an information source. Here, we will focus on how social information use is modulated by peer characteristics pertaining to expertise and social status, which are one of the most important and well-researched attributes of peer influence. These peer characteristics relate to how useful we think the peer information is, or a sense of trust in whether the peer holds accurate beliefs and is willing to share these, depending on their goals. Information from an expert is likely to be more reliable than from a novice leading to more informational social influence exerted by the former, and copying normative behavior displayed by a high versus a low status peer might impact your own social status more, especially when you find yourself transitioning from one social group to another. A demonstrator’s performance or reliability, for example, indeed are important factors for social information use (Morgan et al., 2012, Olsen et al., 2019, Gradassi et al., 2022). In addition to a demonstrator’s objective performance, conveyed performance or subjective confidence statements heavily influence copying behavior, such that more confident people, or people who state that they are more confident, exert more influence over both group and individual decisions (Gradassi et al., 2022 , no date; Zarnoth and Sniezek, 1997; Morgan et al., 2012; Moussaïd et al., 2013), as do more extreme opinions (Price and Stone, 2004, da Silva Pinho et al., 2021). Moreover, decisions of multiple individuals yield a larger impact when they are in high agreement versus when they substantially differ from each other (Molleman et al., 2020). In a similar vein, if you want to fit into a new social group, it would be more useful to act similar to a group member who is high in status relative to someone with low status, suggesting that high status peers exert more normative influence. Indeed, peer characteristics pertaining to social relations and group structure have been identified to influence to what extent these peers influence others’ behavior (Kendal et al., 2018). For example, high school students are more strongly influenced in their perceptual decision making by classmates who are perceived as more popular or who are socially closer (Gradassi et al., 2022). Additionally, decisions or judgements made by groups of people tend to be more influential when that group is larger and when there is higher consensus within that group (Morgan et al., 2012, Moussaïd et al., 2013, Molleman et al., 2020, da Silva Pinho et al., 2021).

### 5.2 Social cognition

**Fig. 4:**
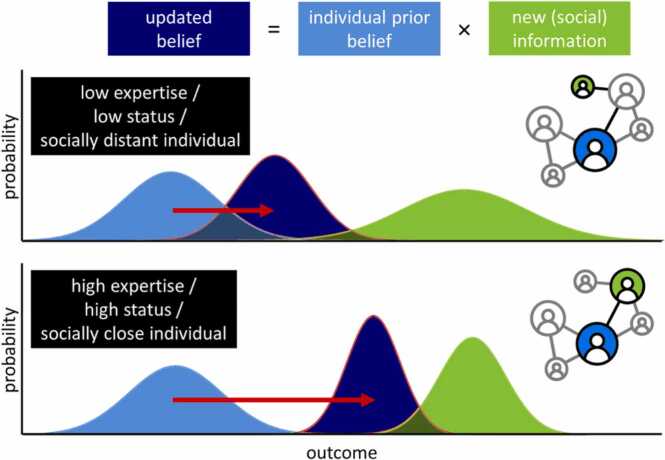
Bayesian updating based on peer characteristics. An agent combines their prior belief or information with new social information to arrive at an updated belief of the expected value or outcome. In the case of a social source with low expertise, low status or who is a loose acquaintance (top panel), the social information will be assigned a lower weight, as depicted by a narrower distribution, than when the social source had high expertise, was of high social status or was a close friend, as depicted by a narrower distribution (bottom panel). This would result in a weaker impact of the same social information (red arrow). Differentiation between how much weight one assigns to different social sources is hypothesized to improve across adolescence, and can happen through explicit knowledge or Theory of Mind.

As described, social information likely is more useful when it comes from a reliable or important source, such as someone with high expertise or social status. This weighting of social information use can be based on explicit social influence from known others with known expertise, status or social distance, such as a recommendation from a close relative (Fig. 4). However, sometimes the other’s expertise or status are not entirely clear. How do we assess the value of the information provided by that other person? How do we assess whether to trust someone and how do we determine their goals and how do these abilities pertaining to social cognition change during adolescence?

#### 5.2.1 Theory of Mind

Oftentimes it is not clear what the benefits and costs of someone else’s action are for them and thus why they decided to perform that action. Their previous actions and the current situation might help you to infer these hidden reasons and goals, also called Theory of Mind (ToM) or inverse RL (Dennett, 1978, Burke et al., 2010, Cheong et al., 2017, Feldmanhall et al., 2018, Jara-Ettinger, 2019, Najar et al., 2020): Rather than a value function determining which action to choose, which elicits an outcome that in turn updates the value function, ToM happens when we infer someone else’s value function using the set of actions they performed and the outcomes they received in the past (Fig. 1, in blue). If we do this in an iterative manner, as with regular RL, we can slowly update our estimate of the other’s goals and beliefs. As with regular RL, such inferences can carry a high level of uncertainty, because different underlying goals can yield similar behavioral actions (Rusch et al., 2020, FeldmanHall and Nassar, 2021). Similarly, due to incomplete information, we can often only infer which individuals possess accurate knowledge or are highly regarded by a particular group. A more Bayesian approach, as with individual learning, would therefore capture a probability distribution over the other’s hidden goals, accuracy or status (Kilner et al., 2007, Baker et al., 2017), to ultimately decide if the informational value of the other’s actions should be used to update your own value function or to decide if you want to use social information. In support of this assumption that people indeed use Bayesian updating to differentiate between different sources, it has been demonstrated using computational modeling that information from peers who are in high agreement with each other was assigned a higher weight resulting in stronger social influence than information from peers who were in low agreement (Molleman et al., 2020). This differential impact of different social sources can be implemented in the social RL framework as a variable that modulates the impact of the informational or normative social information. For example, social information stemming from a more reliable or a high status peer would be translated to a narrower curve, and thus a more pronounced shift of the posterior, than information from a less reliable or a lower status peer (Fig. 4). Therefore, a Bayesian framework is very suitable to incorporate information representing different levels of evidence stemming from different social sources.

### 5.3 Neural mechanisms of Theory of Mind and social cognition

Different types of processing might be involved in the way in which we learn about others in order to learn from others. Simple observational learning to assess the value of information of others or reward structures being learned by others involves the same striatal and vmPFC network, in connection with the ACC, that is also crucial for individual RL, suggesting that both individual and vicarious learning rely on the same computational mechanisms of (e.g. monetary and social) reward values and prediction errors of experienced and observed outcomes (Burke et al., 2010, Hare et al., 2010, Jones et al., 2011, Cooper et al., 2012, Suzuki et al., 2012, Boorman et al., 2013, van den Bos et al., 2013, Ruff and Fehr, 2014, Morelli et al., 2015, Toelch and Dolan, 2015, Zaki et al., 2016, Campbell-Meiklejohn et al., 2017). Similarly, more model-based learning about others, such as whether they are generous in general based on prior-learned behavior (Hackel et al., 2015) and how their actual actions compare to their expected actions (Burke et al., 2010, Suzuki et al., 2012, Morelli et al., 2015) rely on executive control areas including the parietal cortex, dorsomedial PFC and dorsolateral PFC. Although these findings hint at similar mechanisms for both individual and social learning and decision making (Heyes, 2012, Ramsey and Ward, 2020), several brain areas have been found to be particularly involved in social cognition, including the temporo-parietal junction (TPJ) (Saxe and Kanwisher, 2003, Saxe, 2006, Hampton et al., 2008, McKell Carter et al., 2012, Morishima et al., 2012, Rushworth et al., 2013, van den Bos et al., 2013, Hackel et al., 2015, Zaki et al., 2016), the posterior superior temporal sulcus (pSTS) (Pelphrey et al., 2004, Lahnakoski et al., 2012; DV Smith et al., 2014; Deen et al., 2015; Isik et al., 2017), the medial PFC (Amodio and Frith, 2006, Apps and Ramnani, 2017, Denny et al., 2012, Hampton et al., 2008, Saxe, 2006, van Overwalle, 2011) and the (more rostral part of the) anterior cingulate gyrus (Behrens et al., 2008, Jones et al., 2011, Fareri et al., 2012, Chang and Sanfey, 2013, Apps and Ramnani, 2014, Apps et al., 2016, Hill et al., 2016, Zhang and Gläscher, 2020). Notably, the input from this social brain network to the valuation and executive control areas and the functional coupling between them seems to be of paramount importance for the integration of social signals and traditional decision-making processes (Hare et al., 2010, Heyes, 2012, van den Bos et al., 2013; DV Smith et al., 2014; Ruff and Fehr, 2014; Lindström et al., 2018; Zhang and Gläscher, 2020). These findings therefore raise the hypothesis that neural connections between areas associated with ToM capacity and areas associated with confidence judgements largely define one’s ability to discriminate between attributes of different peers.

### 5.4 Developmental changes in the neural mechanisms of social cognition

To form predictions about how different sources affect adolescent-specific social information use, we need to consider the behavioral and neurodevelopmental trajectories of these differentiating abilities during adolescence. Developmental changes in the strength of these connections would modulate the impact of social information (Kilford et al., 2016). Indeed, a more advanced puberty stage as assessed by the level of pubertal hormones correlated with increased functional connectivity between the TPJ and the dorsomedial PFC (Klapwijk et al., 2013) and compared with younger adolescents, older adolescents show increased neural activity in both the TPJ and the dorsolateral PFC, which was associated with increased sensitivity to the perspective of others (Güroǧlu et al., 2011, van den Bos et al., 2011). Moreover, structural changes in the social brain network continue into early adulthood (Mills et al., 2014) and ToM capacity still improves from late adolescence to early adulthood (Choudhury et al., 2006, Dumontheil et al., 2010). These studies suggest that the social brain and its connections with executive control areas continue to develop into late adolescence, making us more adept at inferring others’ goals and mental states and differentiating between different individuals in terms of expertise and status. Such a superior ToM capacity would result in less uncertainty about he usefulness of the information from a particular sources, which in turn would lead to an increased proficiency to strategically and selectively follow useful social information and to ignore unreliable and untrustworthy sources.

### 5.5 Neural representation of social distance

Another recent line of research has started to unravel the differences in neural activation in response to socially close versus distant others. Perceived social distance often relates to how similar we feel to other people, which can be of informational value, since similar others often hold similar values and preferences to ours, which can be used to infer opinions in other domains. For example, you are having dinner with a friend of yours who you know really likes your home-made soup, and now he advises you to try the pasta dish, you might infer you will also like that pasta dish. Conversely, perceived similarity might also evoke normative influence because we particularly want to be liked or accepted by people from within our social group. How do we distinguish between socially close and distant others? The medial PFC, part of the social brain network, has been shown to respond differently when making judgments about close versus unfamiliar others (Krienen et al., 2010) and self-reported social proximity positively correlated with self-other similarity in the medial PFC, such that the people we feel closest to are represented more similarly to the neural representation of ourselves (Courtney and Meyer, 2020). Moreover, activation patterns in the inferior parietal lobule, an area that overlaps with the TPJ, distinguished between judgements of social distance in a similar fashion as they distinguished between spatial and temporal distance (Parkinson et al., 2014, Parkinson et al., 2017). Future studies should test if differences in for example peer expertise or peer confidence parallel the neural patterns as seen for social closeness. So far, developmental changes in these representations of social distance have not been researched. It thus remains an empirical question whether representational distinction in these neural areas would also improve over age, especially during adolescence, as has been demonstrated for the neural and behavioral abilities pertaining to ToM, and whether a sharper neural contrast between close and distant others also translates to larger differences in social influence (see also Fig. 4).

## 6 Summary

Adolescence is a period in which we are generally more sensitive to the influence of our peers than in any other stage of our life. In this review, we aimed to shed light on the neurocognitive mechanisms involved in this process, using the conceptual perspective of Bayesian reinforcement learning in a social context. This comprises value-based decision-making while taking into account the various forms of uncertainty that come with it, including our own uncertainty when we find ourselves in ambiguous or risky situations or when we rely on ambiguous stimuli, but also the uncertainty about the knowledge and intentions of and thereby information from others.

**Fig. 5:**
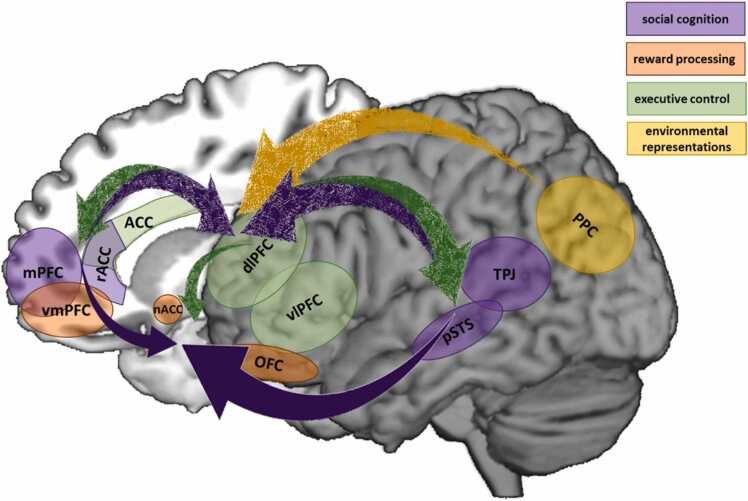
Candidate brain areas comprising the social learning network. Connections between social cognition (purple) and reward processing (orange) areas mediate responsiveness to social stimuli. Reciprocal connections between social cognition and executive control (green) areas modulate this responsiveness by using Theory of Mind to map representations of social stimuli onto representations of subjective confidence. These same executive control areas also map representations of the environment (yellow) onto confidence representations, thereby signaling environmental uncertainty. Confidence signals can then, in an environment-dependent way, be used by the reward processing areas to strategically and selectively determine which social information to incorporate into the action-selection process. Because the executive control system and its connections are still in development during adolescence, the differentiation between different levels of confidence gradually improves from adolescence to adulthood. Abbreviations: TPJ, temporoparietal junction; pSTS, posterior superior temporal sulcus; rACC, rostral anterior cingulate cortex; mPFC, medial prefrontal cortex; OFC, orbitofrontal cortex, nAcc, nucleus accumbens; vmPFC, ventromedial prefrontal cortex; vlPFC, ventrolateral prefrontal cortex; dlPFC, dorsolateral prefrontal cortex; ACC, anterior cingulate cortex; PPC, posterior parietal cortex.

In line with a Bayesian framework, using peer information is more useful in situations of high uncertainty in which priors are very uncertain and where new pieces of information can drastically reduce uncertainty of posterior probability distributions (Toelch et al., 2014). Different studies have yielded seemingly contradictory results with regard to how adolescents’ perceive uncertainty, with adolescents generally being less ambiguity averse than adults, while at the same time showing more information seeking before reaching a decision than adults, being indicative of higher perceived uncertainty. These seemingly contrasting notions might be explained by the relatively new and uncertain environment in which adolescents find themselves during this period of social reorientation and becoming more independent of parents and caretakers. In adapting to this new situation, adolescents might learn and expect the world to be more volatile and uncertain, even in cases when it is quite stable, while at the same time exhibiting lower ambiguity aversion in order to explore and learn about the world. Alternatively, regions of the executive control network that have been demonstrated to be important for both model-based learning and signaling decision-confidence include the posterior parietal cortex, the ACC, the dorsomedial PFC and the dorsolateral PFC (Fig. 5). These are areas that are still in development during adolescence and into early adulthood, possibly leading to less accurate mental models of the current environment and less accurate estimates of confidence. These relatively unreliable confidence estimates are therefore less useful for decision-making, possibly making adolescents more prone to seek out additional information from peers. However, as discussed above, how exactly the later neural development of the executive system relates to accurate tracking of uncertainty and how this affects social information seeking in a strategic way, thus distinguishing between low and high uncertainty situations has yet to be experimentally investigated.

Compared with other age groups, adolescents appear to be more susceptible to normative peer influence, where advice from or observation of peers adds a normative bias to the expected value of that particular behavior, making it more likely to be performed or copied. Although this is usually considered as an undesirable adolescent characteristic, this influence occurs both in the prosocial and antisocial or risk-taking domain, depending on the value adolescents think their peers assign to certain behaviors. The striatum, particularly the ventral striatum or nucleus accumbens, the orbitofrontal cortex and the (ventro)medial PFC, which have traditionally been implicated in value-assignment and value-updating based on differences between actual and expected outcomes experienced in the past (Fig. 5), are particularly responsive to social rewards during adolescence. Furthermore, enhanced connectivity with social and executive brain areas seems to be important to translate social stimuli into the reward function in order to for example enhance social status or avoid social punishment. In this regard, it is important that adolescents are indeed aware of the prevailing norm. Yet, it has been found that adolescents may overestimate the frequency of risky behavior within a group, possibly resulting in a larger influence of risky behavior (Graham et al., 1991, Ciranka and van den Bos, 2021b). Thus, rather than merely directing efforts into minimizing negative social influence, policies could be tuned toward informing adolescents about the true norms (Perkins et al., 2011).

The valuation and executive control areas also seem pertinent for decision-making in a social context. However, input from areas more specific to social cognition, including the temporoparietal junction, the rostral ACC gyrus and the medial PFC appear to incorporate social signals into the learning and decision-making processes (Fig. 5). Although it appears that neural patterns in the temporoparietal junction and medial PFC signal parametric differences between social sources, such as how close we feel toward the other, it is still an open question whether the degree to which one discriminates between different types of social sources is shaped by the connection strength between the social and the executive network. A stronger reciprocal connection between the two networks might enhance the ability to adaptively discriminate between different social sources by accurately estimating the status, trustworthiness and mental-states of others, and thereby the value of the information they provide in a model-based Bayesian way. These value functions associated with different social sources would then be translated to the valuation network to ultimately decide whether and to what extent to use the information stemming from particular social sources (Fig. 4). It might thus be the case that compared with children and adults, adolescents are influenced by their peers to a larger degree due to a highly engaged valuation network and strong connections between the social and the valuation network, but because the executive and social network are still developing, they might have a harder time discriminating between different situations in terms of uncertainty and between different social sources in terms of their trustworthiness.

## 7 Outlook

Many outstanding issues remain, with examples of open questions already alluded to in the respective sections above. Below, we will briefly discuss a set of open issues categorized into the advantage provided by computational models in researching developmental changes, whether neural mechanisms of informational influence are similar to those of normative influence, and how the adolescent surge in social influence might be, at least partially, be a direct results of their changing environment.

### 7.1 Computationally testing developmental changes in social influence

Computational models can help testing how determinants of social influence, such as normative versus informational considerations and uncertainty affect adolescent decision making compared with younger and older age groups. For example, it can be tested how an initial piece of advice from peers affects decision-biases. For instance, in contrast with the integration of purely informational influence, which would only enter the model once to update the expected value function, normative influence would be used at every new decision step, as long as the norm stays relevant (if peers are around or will hear about the decisions made) and there is no need to update the estimated social status of the other. For example, when a high status person, who is a vegetarian, tells you that you should order the vegetarian soup, you might update your value function for this soup in such a way that, on that occasion, that soup receives a higher expected value than the other soups and you will thus order it. This could have been brought about by either informational or normative influence. Next, if it turns out that you do not like the soup and you are particularly affected by informational influence, this will elicit a strong negative prediction error and the next time you will visit the same restaurant with the same person, you might be less inclined to order that same vegetarian soup again. In contrast, if you are someone who is particularly affected by normative influence, the combined low informational (taste) but high social value results in a smaller prediction error and you might still order the soup the next time to stay in the other’s good graces, even if that means that you will lose out on taste. The impact of the normative value might also depend on the context, such that in a more normative-oriented task, for example a task in which the peer is a known acquaintance or close friend of the participant, or a task in which the participant is observed by the advice-giving peer, the normative bias compared with the informational effect on the prior might be stronger than in a purely information-oriented task.

**Fig. 6:**
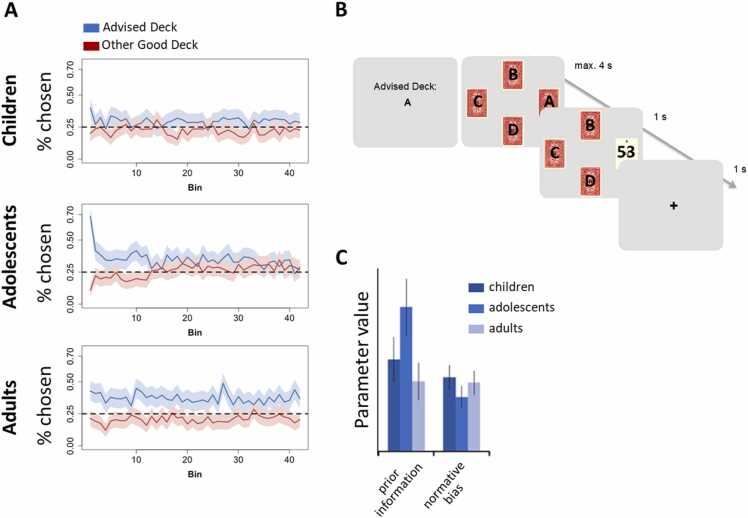
Modeling social learning. A. Depiction of how the percentage of participants’ choices of the advised good deck (blue) and the other, non-advised, good deck of cards (red) changes over the course of the task. Choices are displayed separately for children, adolescents and adults. B. Participants played a learning task in which they could choose between four decks of cards, two of which were associated with a higher average payout (good decks) and two of which were associated with a lower average payout (bad decks). The goal of the game was to maximize cumulative rewards. They were first shown which of the four decks was advised by a previous player. They then played 210 rounds in which they chose one of the decks and received feedback about their payout. **C.** All age groups showed a significant effect of the advice at the start of the experiment (prior information), such that they opted the advised deck more often than the other decks. All age groups also exhibited a normative bias, such that they showed a consistent preference for the advised deck over time. However, only prior information showed an effect of age, such that adolescents initially relied more strongly on the advice than children and adults.

A follow up step would be to test whether choice parameters, such as informational value, normative value and their respective prediction errors, show developmental trends. For example, a recent study used computational modelling to investigate developmental differences in learning from an initial piece of peer advice (Rodriguez Buritica et al., 2019). They found that, whereas all age groups showed a normative bias toward the advised option across the entire task, adolescents put more weight on peer advice at the beginning of the learning task (their priors; Fig. 6), showing that adolescents might initially be more sensitive to peer influence, this effect decreases as new information comes in. The next step is to investigate whether such computationally derived parameters correlate with neural patterns or activation, for example in reward-related areas such as the ventral striatum and ventromedial PFC or their connection with the TPJ.

Figure adapted from Rodriguez Buritica et al. (2019).

### 7.2 Neural mechanisms of conformity

Hitherto, it is unclear whether from a neurocognitive perspective, normative influence is mechanistically similar to informational influence or not. There have been studies showing that conforming with a group after discovering that your private opinions and that of the group are in conflict, correlates with activity in the posterior medial frontal cortex and striatum (Klucharev et al., 2009, Klucharev et al., 2011, Berns et al., 2010, Campbell-Meiklejohn et al., 2010), which was interpreted as a form of performance-monitoring sharing the same neural mechanisms as regular, non-social reinforcement learning mechanisms. However, a later study indeed found overlapping activity in the posterior medial frontal cortex and striatum for social conformity effects and regular non-social prediction error processing, but the patterns of activation, as determined using multivariate pattern analysis, distinctively differed between the two processes (Levorson et al., 2020), suggesting that they rely on different neural mechanisms. This would intuitively be in line with how they affect the decision-making process, with informational and normative information affecting accuracy-based and social value, respectively, and only the latter not always being relevant in every context. Therefore, only informational but not normative influence is needed to be incorporated into a personal model of reality. This could be more directly investigated from a neuroscientific perspective by testing whether, in the same paradigm, informational social cues that vary parametrically in value or importance elicit different patterns of neural activation or connectivity than normative social cues.

### 7.3 Environment-driven adolescent surge in social influence?

As we have discussed, adolescents find themselves in a rapidly changing environment, which can be experienced as very uncertain and asks for exploration and information seeking. It has been postulated that this environment alone, rather than any adolescent-specific changes in neurocognitive computations such as heightened engagement of the valuation network or maturing connections between networks, can partly capture the increase in social information use during adolescence (Ciranka and van den Bos, 2021a). The inherently risky act of exploring the environment decreases the uncertainty associated with the novel environment and relying on advice from peers might speed up this process, making it very adaptable during adolescence. The question thus arises how a changing environment drives adolescent-specific behavior, possibly mediated by shifts in neurochemical transmission in response to these changes (Segovia et al., 2010, Egerton et al., 2017).

## 8 Conclusion

Throughout adolescence, social and developmental changes take place that shape the way in which they use social information or learn from their peers. Structural and functional changes in the brain’s valuation, executive control and social cognition networks, as well heightened uncertainty and sensitivity to social relationships due to social reorientation during this period make adolescence a period of increased peer influence. Both informational and normative social information seem to affect adolescents’ decision-making to a larger degree than younger or older people. This is often viewed as a negative inclination, especially when the influence is of a normative nature, which is grounded in a dual-systems theory that postulates heightened socio-emotional responsiveness combined with immature impulse control in adolescence (Casey et al., 2008, Steinberg, 2008, Shulman et al., 2016). However, many instances of social influence comprise positive peer influence, such as enhanced effort or prosocial behavior, which are hard to reconcile with the dual-systems notion of immature self-regulation, raising the need for complementary models (Pfeifer and Allen, 2012, Gladwin and Figner, 2014). Here, we put forward a unifying perspective of Bayesian social learning to explain both negative and positive social influence, which combines reward-based decision-making in both the informational and the social domain. Relying on others and copying their behavior in order to maximize both material and social rewards might well be very adaptive during a period in which one needs to reestablish their identity and model of the world within a new and uncertain social environment (Crone and Dahl, 2012). The adolescent development of social learning relies on maturing neural connections between social cognitive, executive control and reward areas (Fig. 4). This enhances the ability to strategically discriminate between different levels of uncertainty and different social sources to determine to what extent to use their information, thereby accommodating ToM into the framework of social influence. Future computational studies that closely examine which neural areas and connections are involved in this selective sensitivity will give us more insight into how exactly the informational and normative factors are orchestrated and integrated.

## Declaration of Competing Interest

The authors declare that they have no known competing financial interests or personal relationships that could have appeared to influence the work reported in this paper.
